# Supplementary material for: Complexome profiling of the Chlamydomonas psb28 mutant reveals TEF5 as an early PSII assembly factor
Source: Plant Cell. 2025 Mar 17;37(6):koaf055. doi: 10.1093/plcell/koaf055 (PMC12164590; doi:10.1093/plcell/koaf055)
Supplement: koaf055_Supplementary_Data [file koaf055_supplementary_data.zip › psb28_interactive.zip!/plots/intensity/qualityPlots/peptides/OTU4_Cre06.g274700.t1.1.html]

### Cre06.g274700.t1.1

Trivial name: OTU4  
SeqCoverage [median]: 10.50  
Mass: 44.12 kDa  
  
 

| Sequence | MissedCleavage | Mass [Da] | wtIntensity | muIntensity | Score |
| --- | --- | --- | --- | --- | --- |
| AVRPSSASGTSTK | 1 | 1247.65 | 339.19 | 561.83 | 72.68 |
| NVVAQQVSGDR | 0 | 1171.59 | 310.18 | 674.91 | 132.01 |
| SAAAASTAAGAAK | 0 | 1046.54 | 231.69 | 520.23 | 68.89 |
|  |  |  | 881.1 | 1757.0 |  |
